# Electrical Properties of Double-Sided Polymer Surface Nanostructures

**DOI:** 10.1186/s11671-019-3071-2

**Published:** 2019-07-11

**Authors:** Man Zhang, Liangping Xia, Suihu Dang, Axiu Cao, Lifang Shi, Hui Pang, Chunlei Du

**Affiliations:** 1 School of Electronic Information Engineering, Yangtze Normal University, Chongqing, 408100 China; 2 Institute of Optics and Electronics, Chinese Academy of Sciences, Chengdu, 610209 China; 3 Chongqing Key Laboratory of Multi-Scale Manufacturing Technology, Chongqing Institute of Green and Intelligent Technology, Chinese Academy of Sciences, Chongqing, 400714 China

**Keywords:** Double-sided nanostructures, Nanoimprint lithography, Electrical properties, Open-circuit voltage, Short-circuit current, Pressure force

## Abstract

In this study, double-sided polymer surface nanostructures are fabricated using twice nanoimprint lithography and metal deposition technique. We perform electrical property measurement on these double-sided surface nanostructures. Open-circuit voltage and short-circuit current of the as-prepared samples with double-sided surface nanostructures and conductive electrode are recorded using an oscilloscope with applying different external force. The measurements are carried out at room temperature. We find that the intensity of open-circuit voltage and short-circuit current for the double-sided surface nanostructures depends strongly on the sizes, shapes, and arrangements of nanostructures and pressure force. The strongest electrical property can be observed in the hexagon nanopillar arrays with the diameter of about 400 nm containing sub-50-nm resolution sharp structures at the force of about 40 N. We discuss the physical mechanisms responsible for these interesting research findings. The experimental results we study are relevant to the applications of double-sided surface nanostructures such as a nanogenerator, pressure sensors, and nano-optoelectronic devices.

## Background

Nanostructures on surfaces attract much interest as an efficient media for surface-enhanced Raman scattering (SERS), surface plasmon resonance, nonlinear optical and electrical response, and plasmonic excitation such as nanoparticles, nanograting, and nanopillars, especially metal surface nanostructures [1–5], which have potential applications as electronic, magnetic, photonic, optoelectronic, and sensor devices [6–10]. From a viewpoint of physics, the basic physical properties of surface nanostructures differ significantly from those of bulk materials with the same components. In particular, surface effects can be observed in the surface nanostructures. Therefore, surface nanostructures have been a major focus of research on surface materials which can be taken as a fundamental building block of nanotechnology and nanodevices. It should be noticed that polymer surface nanostructures have displayed unique optoelectronic and electrical properties due to the triboelectric effect that is electrostatic induction occurring within polymer materials [11–13]. Nanoscale structures increase surface roughness and the contact friction area to enhance the triboelectric effect, especially double-sided surface structures. The triboelectric effect in surface nanostructures can cause the generation of large electrical charges, which can obtain current by connecting electrodes and wires. The triboelectric effect in polymer surface nanostructures and related phenomena contributes greatly to their promising applications in nanogenerators, pressure and temperature sensors, and other electronic devices [14–17]. The nanogenerators can transfer mechanical energy into electric energy, and the pressure or temperature sensors can transform different pressure or temperature to detectable electrical or optical signals.

As the rapid development of nanotechnology, it is now easy to fabricate periodic and complex unordered surface nanostructures, for example, photolithography, nanoimprint lithography (NIL), self-assembly, and interference lithography [18–22]. As one popular replication nanotechnology, NIL is simple, low-cost, high-resolution, and high-throughput, which is ideal for fabricating polymer nanostructures [23–25]. One major advantage to apply surface nanostructures as electronic devices is that the electrical response of the surface nanostructures can be tuned and modulated via varying structure parameters such as diameter, shape, and arrangement of nanostructures. Therefore, it is of importance and significance to examine basic electrical properties of surface nanostructures.

In this article, we present a detailed experimental study on the electrical properties of two kinds of double-sided surface nanostructures, such as grating and nanopillar arrays. The double-sided polymer surface nanostructures are fabricated using twice NIL process. Because the nanostructures on two side surfaces need not be aligned, the imprinting process is simple and low cost. The conductive electrode for measuring electrical signals is prepared by the metal deposition technique, such as indium tin oxide (ITO) or Ag film. We would like to research how these surface nanostructures can respond to external pressure, how their electrical properties depend on the sample’s parameters, and how the open-circuit voltage and short-circuit current of the as-prepared samples change.

## Methods

### Samples

**Fig. 1:**
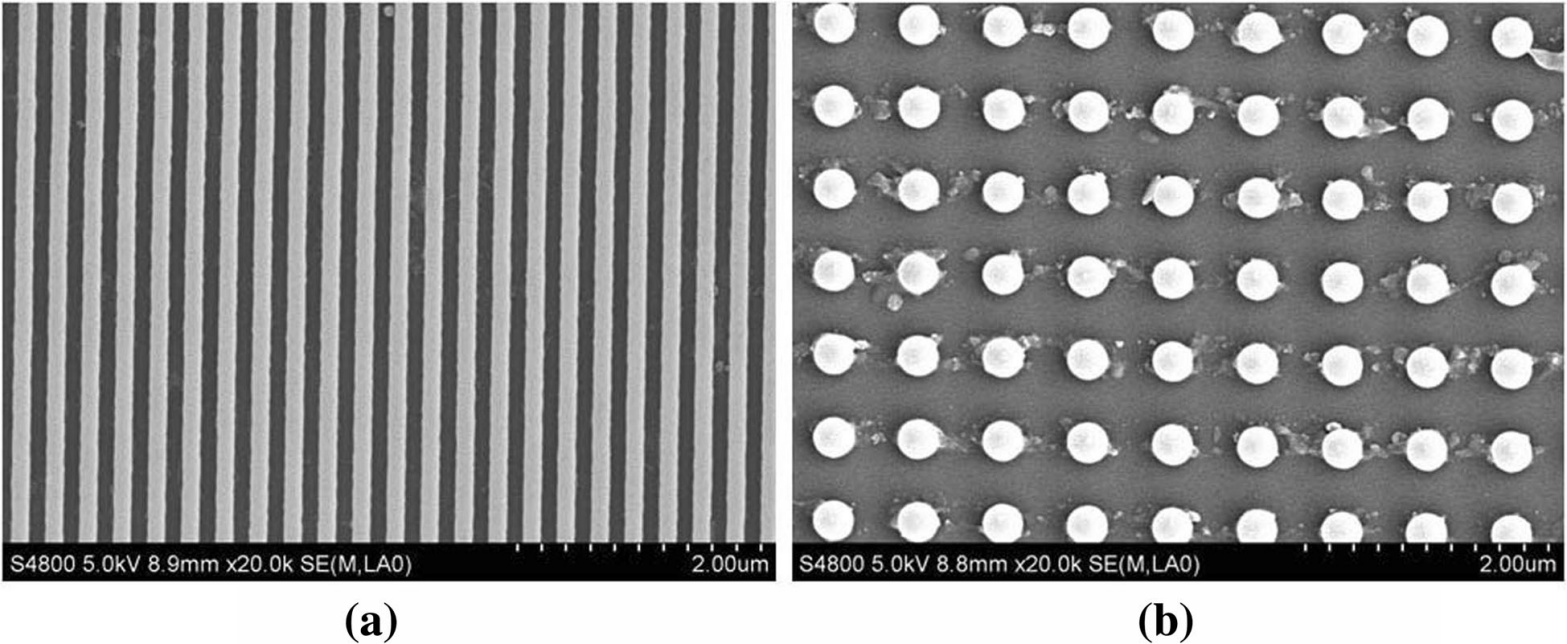
SEM images of two kinds of surface nanostructures. A grating (**a**) and a nanopillar array (**b**) are shown

In this study, two kinds of surface nanostructures to be measured are fabricated such as grating and nanopillar arrays, and the scanning electron microscopy (SEM) images are shown in Fig. 1. The period of the grating is about 300 nm, the width is about 160 nm, and the diameter of nanopillar is about 300 nm.

**Fig. 2:**
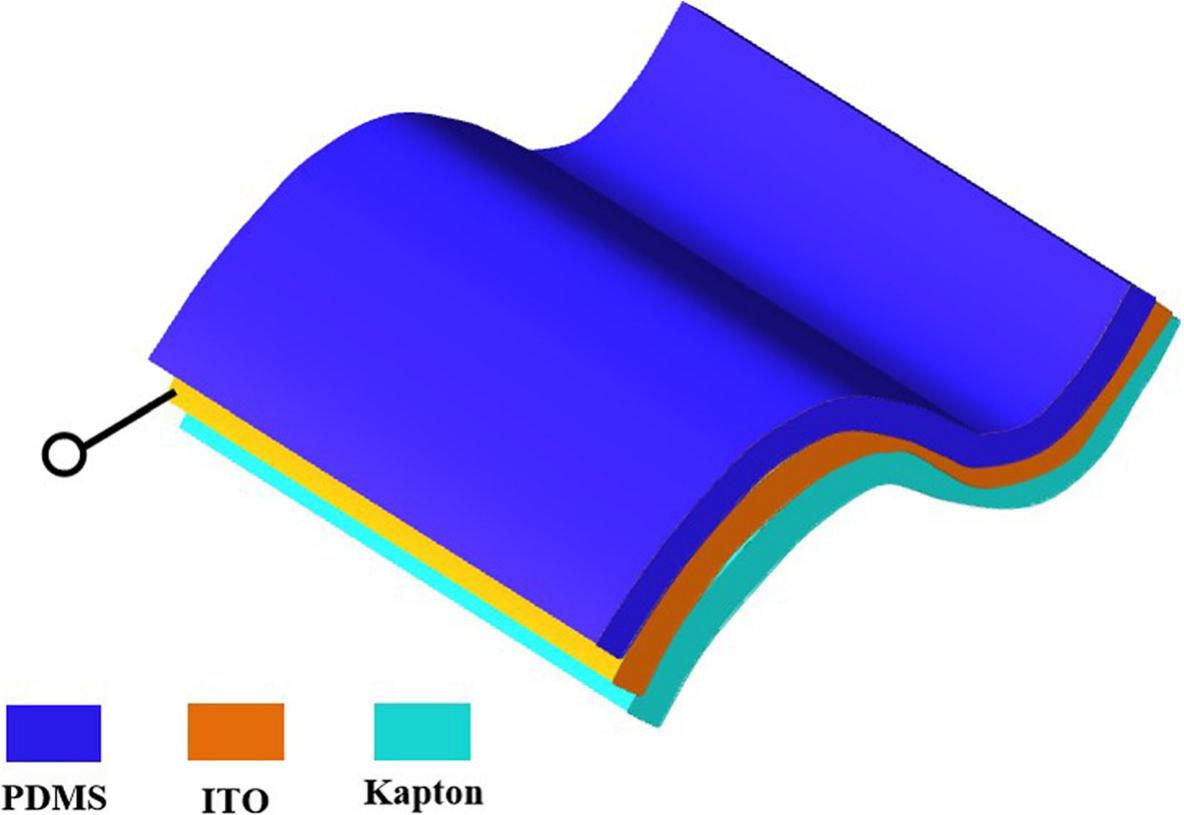
Schematic of the double-sided polymer nanostructures

The prepared samples with double-sided surface structures are fabricated by combining twice UV-curable NIL, and the conductive electrode layer between the double-sided structures are prepared by electrodeposition of ITO film. The schematic of the double-sided polymer nanostructures is depicted in Fig. 2. The double-sided structure materials are polydimethylsiloxane (PDMS) and Kapton that are elastic materials. The intermediate layer is a thin ITO film; thus, the integrated device is flexible. The electricity signal is generated due to the coupling effect of contact electrification and electrostatic induction during contact-pressure-separation operation, which is the principle of measuring electrical properties of the double-sided surface nanostructures.

When deformed by an external-touched mechanical pressure deformation provided by other materials, triboelectric charges are generated and distributed on the polymer surfaces. As soon as the deformation starts to be released, the external-touched materials become separated with the polymer surface. These triboelectric charges cannot be compensated, leading to induce opposite charges on the ITO electrode to drive free electrons to flow from the ITO electrode to the external circuit. This electrostatic induction process can give an output voltage/current signal.

### Measurement Method

**Fig. 3:**
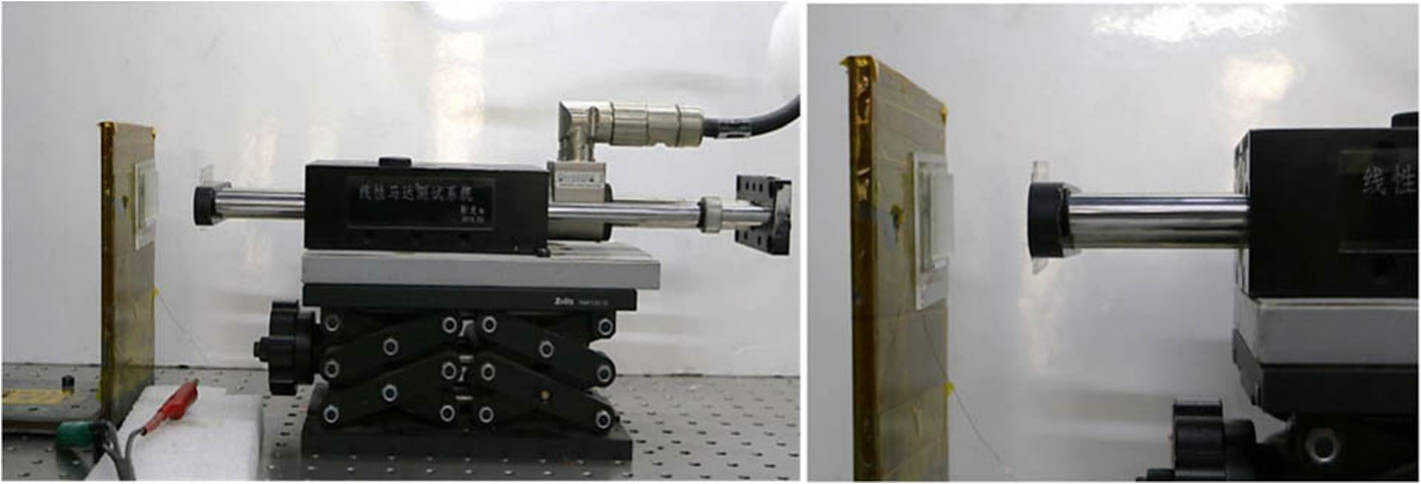
Photograph of experiment setup applying external force

For the measurement of electrical properties of three kinds of surface nanostructures with different sizes, patterns, and arrangements, the measurements are carried out under the external force within 0.5~50 N provided at room temperature in Fig. 3. The electrical properties are recorded using the adjustable linear motor (E1100-RS-HC), current and voltage test device (Keithley 6514), low noise amplifier (Stanford SR570), and oscilloscope (MDO 3014). The change of force is achieved in the adjustable linear motor, and the oscilloscope could measure the voltage and current curve. The experiment setup applying pressure force onto the samples’ surfaces is shown in Fig. 3.

## Results and Discussion

**Fig. 4:**
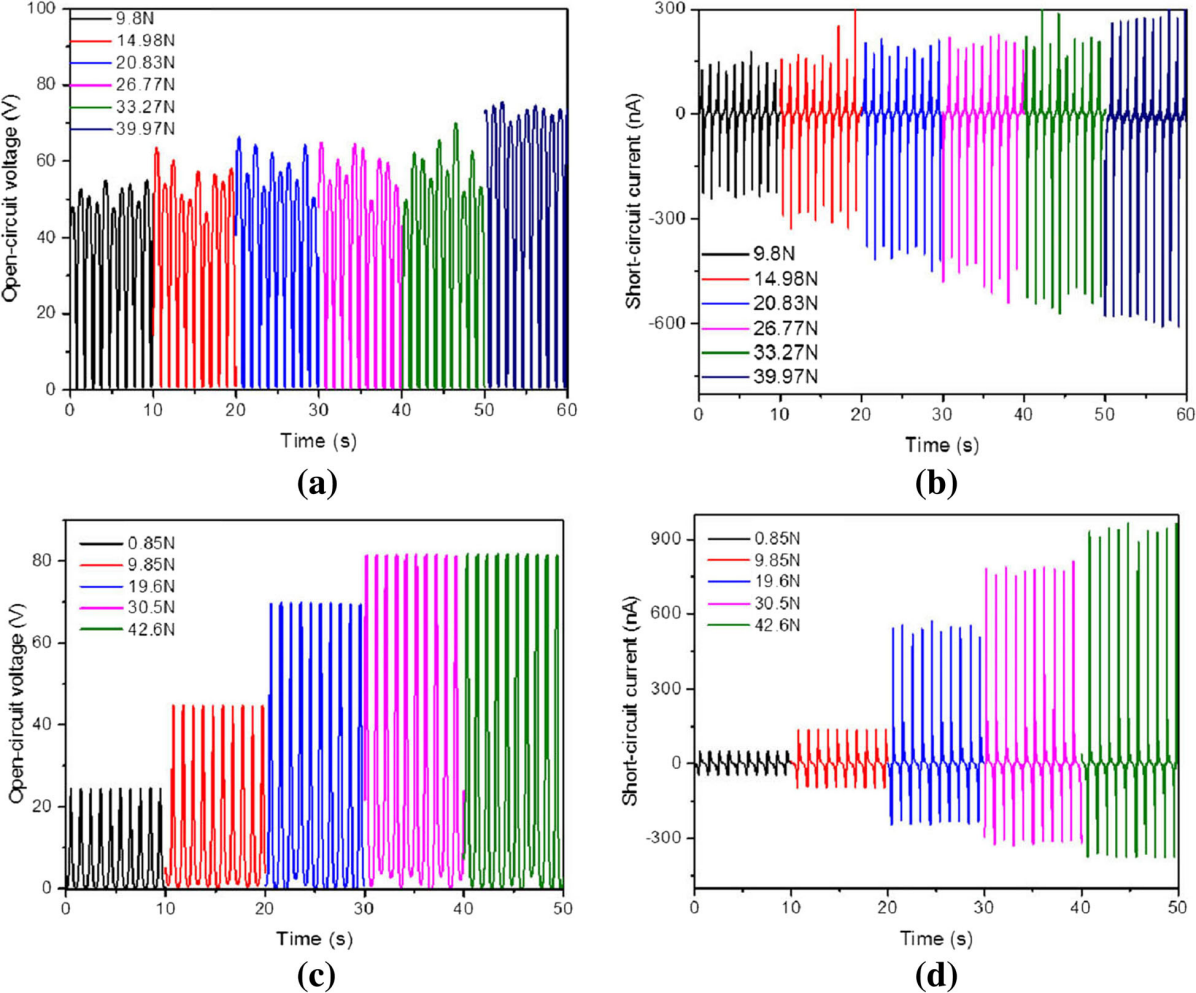
The electrical properties for surface nanostructures. The results for grating (**a**, **b**) and a nanopillar array (**c**, **d**) are shown

The electrical properties for different surface nanostructures are shown in Fig. 4 at different external pressure force. The output open-circuit voltage and short-circuit current of the grating and nanopillar arrays are shown in Fig. 4. As can be seen, the intensity of electric properties in surface nanostructures depends strongly on the pressure force. And a similar phenomenon can be found for the grating and nanopillar arrays. The open-circuit voltage and short-circuit current changes with the pressure force within 10 s are measured. The measurement results indicate that the electrical properties for grating and nanopillar arrays show different force dependence. The open-circuit voltage of the grating structure increases slowly with the force, but the short-circuit current increases obviously with the force, as shown in Fig. 4a and b. In contrast, the electrical properties of the nanopillar arrays show better, because both open-circuit voltage and short-circuit current increase significantly with the pressure force during the same time, as shown in Fig. 4c and d. However, the open-circuit voltage does not change when the force increases from 30.5 N to 42.6 N, yet the short-circuit current is still increasing. Therefore, the experimental results show that the complicated two-dimensional nanopillars have better electrical performance than one-dimensional grating structures.

**Fig. 5:**
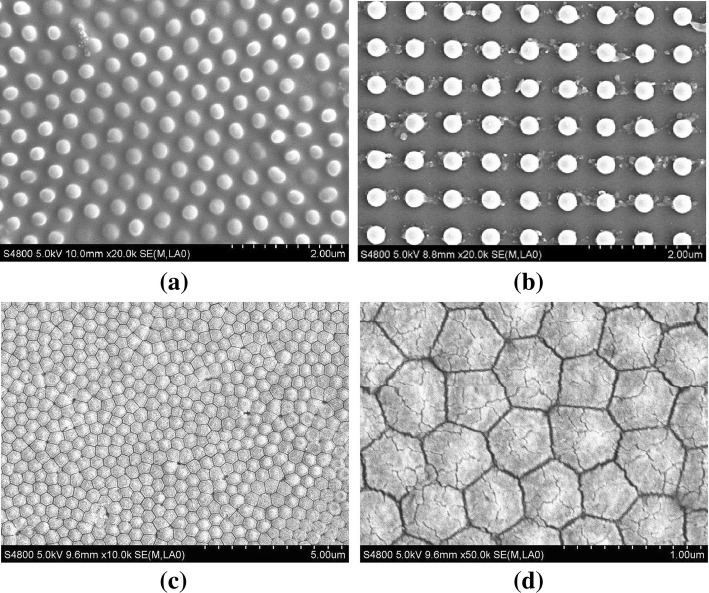
SEM images of three nanopillar arrays. Random (**a**) and square arrangement (**b**) circular nanopillars, hexagon arrangement and shape nanopillar arrays (**c**), and magnification image of hexagon nanopillars (**d**) are shown

To further analyze the electrical properties of nanopillar arrays, different arrangements and shapes of nanopillars are measured such as random, square, and hexagon, and the SEM images of different nanopillar arrays are shown in Fig. 5. The random and square arrangement nanopillars are sparsely distributed in Fig. 5a and b, and the diameters of circular nanopillars are about 300 nm and 400 nm, respectively. The hexagon arrangement and shape nanopillars with about 400 nm of diameter are closely packed in Fig. 5 c. The magnification of one segment of hexagon arrangement nanopillars is shown in Fig. 5d. There is a sharp tip on the top of the nanopillar and a sub-50-nm resolution nanogap between nanopillars, which is similar to the nanoscale pyramid feature.

**Fig. 6:**
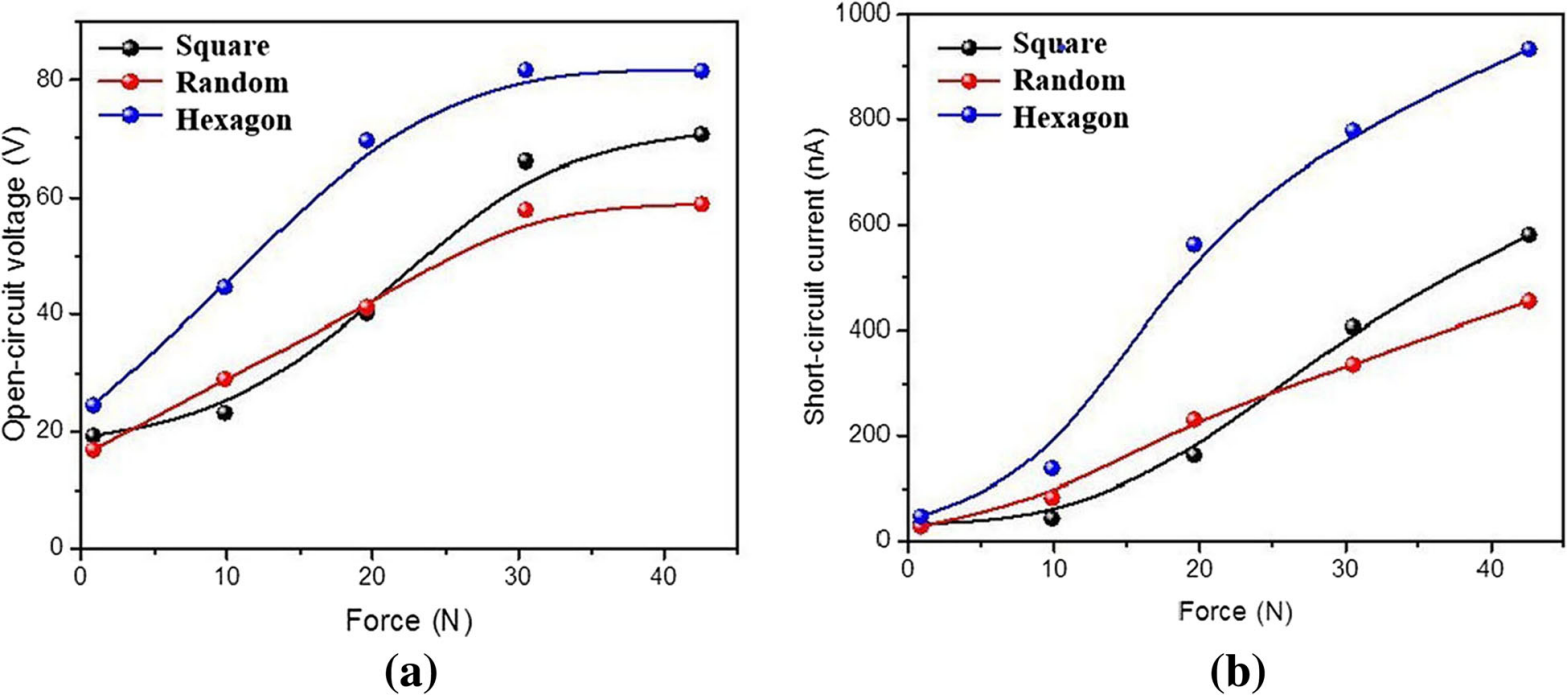
The electrical properties for three kinds of nanopillar arrays, such as open-circuit voltage (**a**) and short-circuit current (**b**)

The electrical performance curves with the force for the different nanopillars samples are shown in Fig. 6. The black, red, and blue curves represent the square, random, and hexagon arrangement nanopillars, respectively. The results indicate that the open-circuit voltage and short-circuit current for three kinds of nanopillars increase rapidly with the pressure force. In contrast, the hexagon arrangement and shape nanopillar arrays show the strongest increase (blue curve) and the electrical properties are the best. When the force is less than 20 N and 25 N, the open-circuit voltage and short-circuit current of the random nanopillars (red curve) are more than that of square arrangement nanopillar arrays (black curve), and the situation is in return as the force continues increasing. One major reason is that hexagon arrangement can provide maximum surface roughness and friction contact area, which contains higher resolution (sub-50 nm) sharp tips and gaps similar to the pyramid feature. Here, the surface roughness is different from the parameter for wafer surface smoothness characterization, which depends mainly on the feature size. Though the diameter of the hexagon nanopillars is similar to others, the sub-50-nm gaps, sharp edges, and corners increase the surface friction roughness and contact area to increase the electrical power output. We find that when the force is greater than 35 N, the open-circuit voltage curves become smooth as shown in Fig. 6a, yet the short-circuit current for three kinds nanopillars are still increasing as shown in Fig. 6b. This indicates that the electrical properties continue to increase with the force, and the increase will become gentle when the force is more than about 40 N.

The experimental results demonstrate that the external pressure force of about 40 N is an appropriate force for the hexagon nanopillar arrays to enhance electrical properties, because too much pressure force may destroy the nanostructure samples. This study can provide a basis for further investigation into other electrical or optical properties.

In this article, the samples with double-sided surface nanostructures are measured. The measuring mechanism of the electrical properties of surface nanostructures indicates that the double-sided surface nanostructures show better electrical performance.

## Conclusions

In this study, double-sided polymer grating and nanopillar arrays have been fabricated using state-of-the-art nanotechnology. The electrical property measurements on these surface nanostructures have been carried out with applying external force at room temperature. We have found that the electrical signal of these samples depends strongly on force and structure arrangements and shapes. In particular, the strongest electrical signal can be observed in the hexagon nanopillar arrays with a diameter of about 400 nm containing sub-50-nm resolution sharp structures compared with other samples. And the appropriate force for measurement of electrical properties is about 40 N. These results indicate that the electrical properties can drive surface nanostructures for the applications in pressure sensor, nanogenerator, and electronic devices. We hope that the interesting experimental finding from this study can provide an in-depth understanding of electrical properties of grating and nanopillars with different arrangements.

## Data Availability

All data generated or analyzed during this study are included in this published article [and its supplementary information files].

## Abbreviations

ITO: Indium tin oxide
NIL: Nanoimprint lithography
PDMS: Polydimethylsiloxane
SEM: Scanning electron microscopy
SERS: Surface-enhanced Raman scattering

## References

[1] Zhang CP, Yi PY, Peng LF, Lai XM, Chen J, Huang MZ, Ni J (2017). Continuous fabrication of nanostructure arrays for flexible surface enhanced Raman scattering substrate. Sci Rep-UK.

[2] Sung MJ, Ma YF, Chau YF, Huang DW (2010). Surface plasmon resonance in a hexagonal nanostructure formed by seven core shell nanocylinders. Appl Optics.

[3] Butet J, Martin OJ (2014). Fano resonances in the nonlinear optical response of coupled plasmonic nanostructures. Opt Express.

[4] Cavas M (2014). Alahmed Z A, Albrithen H A, Yakuphanouglu F: Photoresponse and electrical properties of Al/nanostructure NiFe 2 O 4 /p-Si/Al photodiode. J Electroceram.

[5] Wang MS, Gao CB, He L, Lu QP, Zhang JZ, Tang C, Zorba S, Yin YD (2013). Magnetic tuning of plasmonic excitation of gold nanorods. J Am Chem Soc.

[6] Zhang T, Su D, Li RZ, Wang SJ, Shan F, Xu JJ, Zhang XY (2016). Plasmonic nanostructures for electronic designs of photovoltaic devices: plasmonic hot-carrier photovoltaic architectures and plasmonic electrode structures. J Photon Energy.

[7] Kong YH, Liu XH, Fu X, Li AH (2018). Lateral shift in a spin-orbit-coupling modulated magnetic-barrier nanostructure. Philos Mag.

[8] Zuo J, Li Q, Xue B, Li C, Chang Y, Zhang Y, Liu X, Zhang H, Kong X (2017). Employing shells to eliminate concentration quenching in photonic upconversion nanostructure. Nanoscale.

[9] Ramos M, Barbosa HMC, Correia HMG (2011). The influence of nanostructure on polymer-based optoelectronic device functioning: a computer simulation study. Mater Sci ENG B-ADV.

[10] Linganiso EC, Rodrigues R, Mhlanga SD, Mwakikunga BW, Coville NJ, Hümmelgen IA (2013). GaN nanostructures-poly(vinyl alcohol) composite based hydrostatic pressure sensor device. Mater Chem Phys.

[11] Wang S, Long L, Zhong LW (2012). Nanoscale triboelectric-effect-enabled energy conversion for sustainably powering portable electronics. Nano Lett.

[12] Pan S, Zhang Z (2018). Fundamental theories and basic principles of triboelectric effect: a review. Friction.

[13] Yang X, Daoud WA (2016). Nanogenerators: triboelectric and piezoelectric effects in a combined tribo-piezoelectric nanogenerator based on an interfacial ZnO nanostructure. Adv Funct Mater.

[14] Mohammadpour R (2017). Flexible triboelectric nanogenerator based on high surface area TiO2 nanotube arrays. Adv Eng Mater.

[15] Zhang C, Tang W, Pang YK, Han CB, Wang ZL (2015). Active micro-actuators for optical modulation based on a planar sliding triboelectric nanogenerator. Adv Mater.

[16] Uddin ASMI, Chung GS (2016). A self-powered active hydrogen gas sensor with fast response at room temperature based on triboelectric effect. Sensor Actuat B-Chem.

[17] Garcia C, Trendafilova I, Villoria RGD, Rio JSD (2018). Self-powered pressure sensor based on the triboelectric effect and its analysis using dynamic mechanical analysis. Nano Energy.

[18] Rühe J (2017). And there was light: prospects for the creation of micro- and nanostructures through maskless photolithography. Acs Nano.

[19] Song GJ, Li XR, Li PD, Peng Z, She XL, Li JJ, Sun J (2012). Assembly of one-dimensional polymer nanostructure arrays by photolithographic approaches. J Porous Mat.

[20] Xie GY, Zhang J, Zhang YY, Zhang YY, Zhu T, Liu ZF (2009). Fabrication of metal suspending nanostructures by nanoimprint lithography (NIL) and isotropic reactive ion etching (RIE). Sci China Technol SC.

[21] Wang Y, Zhou W (2010). A review on inorganic nanostructure self-assembly. J Nanosci Nanotechno.

[22] Castro-Hurtado I, Tavera T, Yurrita P, Pérez N, Rodriguez A, Mandayo GG, Castańo E (2013). Structural and optical properties of WO 3 sputtered thin films nanostructured by laser interference lithography. Appl Surf Sci.

[23] Ahn SH, Guo LJ (2009). Large-area roll-to-roll and roll-to-plate nanoimprint lithography: a step toward high-throughput application of continuous nanoimprinting. Acs Nano.

[24] Guo LJ (2010). Nanoimprint lithography: methods and material requirements. Adv Mater.

[25] Ahn J, Kwon S, Jung S, Lee WS, Jeong J, Lim H, Shin YB, Lee J (2018). Fabrication of pyrrole-based electrochemical biosensor platform using nanoimprint lithography. Adv Mater Interfaces.

